# Identification of genes involved in exoprotein release using a high-throughput exoproteome screening assay in *Yersinia entomophaga*

**DOI:** 10.1371/journal.pone.0263019

**Published:** 2022-01-25

**Authors:** Marion Schoof, Maureen O’Callaghan, Campbell R. Sheen, Travis R. Glare, Mark R. H. Hurst

**Affiliations:** 1 Bio-Protection Research Centre, Lincoln University, Lincoln, Christchurch, New Zealand; 2 AgResearch, Forage Science, Lincoln Research Centre, Christchurch, New Zealand; 3 Protein Science and Engineering, Callaghan Innovation, Christchurch, New Zealand; Western Michigan University, UNITED STATES

## Abstract

Bacterial protein secretion is crucial to the maintenance of viability and pathogenicity. Although many bacterial secretion systems have been identified, the underlying mechanisms regulating their expression are less well explored. *Yersinia entomophaga* MH96, an entomopathogenic bacterium, releases an abundance of proteins including the Yen-Tc into the growth medium when cultured in Luria Bertani broth at ≤ 25°C. Through the development of a high-throughput exoproteome screening assay (HESA), genes involved in MH96 exoprotein production were identified. Of 4,080 screened transposon mutants, 34 mutants exhibited a decreased exoprotein release, and one mutation located in the intergenic region of the Yen-Tc operon displayed an elevated exoprotein release relative to the wild-type strain MH96. DNA sequencing revealed several transposon insertions clustered in gene regions associated with lipopolysaccharide (LPSI and LPSII), and *N*-acyl-homoserine lactone synthesis (quorum sensing). Twelve transposon insertions were located within transcriptional regulators or intergenic regions. The HESA will have broad applicability for identifying genes associated with exoproteome production in a range of microorganisms.

## Introduction

Bacteria secrete proteins into the extracellular milieu to interact with the environment as a result of external or internal stimuli. Protein secretion over the protective lipid bilayer in Gram-negative bacteria occurs through various mechanisms ranging from simple protein channels and pores through to more complex multicomponent secretion systems, including the Type 1–9 secretion systems (T1-9SS) [1, 2] and a recently suggested T10SS [3]. Indirect mechanisms such as cell lysis [4, 5] or the release of membrane vesicles (MV) [6–8] have also been reported. Secretion systems are often specific to particular proteins and are triggered under certain conditions. While many secretion systems are well characterized, studies examining the mechanisms underpinning global protein secretion are limited to bioinformatic [9–11] or transcriptomic [12] assessments. Secretion assays are typically tailored to a specific protein of interest and involve the use of antibody-based detection methods such as enzyme-linked immunosorbent assays (ELISAs) or Western blotting [13–15]. Alternative strategies include indirect methods, such as assessment of bacterial mutants for alterations in the secretion of a range of degradative enzymes, including proteases, lipases, and chitinases, using agar plate-based halo assays [16–18]. Other methods are tailored to certain secretion pathways where, for example, TnPhoA reporters were used to detect Type 1 secretion proteins [19, 20]. High-throughput methods, such as 2-D gel electrophoresis [21, 22] and mass spectrometry [23] have been used to characterize the entire exoproteome. However, there are currently no assays allowing the identification of genes involved in the release of exoproteins.

The entomopathogenic bacterium *Yersinia entomophaga* MH96 (Yersiniaceae) is active against a wide range of economically important insect pests, including species belonging to the orders Coleoptera, Lepidoptera, and Orthoptera [24]. In Luria Bertani (LB) broth (Miller) at ≤ 25°C, MH96 releases a wide range of proteins, including the main insect-active toxin complex (Tc), Yen-Tc [25]. *In silico* assessment of the MH96 genome identified a range of putative secretion systems likely to play a role in insect virulence [26]. These include two Type 3 secretion systems (T3SSY1 and T3SSY2), cell-surface adhesion proteins such as fimbria or pili, a T2SS, and a T4SS [26]. In addition to the Yen-Tc, the MH96 genome encodes a range of putative toxins (Vip2, CdtB, LopT, PirAB), as well as degradative enzymes such as proteases, chitinases, esterases, and lipases [26] that must pass through the cell membrane to be effective.

The regulation of exoprotein release can be triggered by contact with host organisms and/or environmental factors, including temperature, pH, or stress factors such as oxygen or nutrient deprivation [27, 28]. Tight regulation of gene expression in response to these stimuli allows adaption and reaction to environmental changes. In this study a High-throughput Exoproteome Screening Assay (HESA) was developed to identify genes involved in the release of proteins into the extracellular matrix. Herein the exoproteome is defined as the concentration and content of proteins released via active transport using secretion systems such as T1- 9SS and MVs, proteins indirectly released by cell lysis [29]. Using this assay, a *Yersinia entomophaga* region of exoprotein release (YeRER) was identified.

## Materials and methods

### Bacterial cultures

All strains used and constructed in this study are listed in S1 Table of S1 File. *Escherichia coli* strains were grown at 37°C, while MH96 strains were grown at 25°C in Luria Bertani (Miller base) broth or on LB agar plates. Unless stated, cultures were incubated with shaking at 250 rpm in a Ratek model OM11 orbital incubator. The *E*. *coli* strain ST18 culture medium was supplemented with 50 μg/mL 5-aminolevulinic acid. The following antibiotic concentrations were used (μg/mL) for each bacterium: MH96 transposon mutant strains kanamycin (100); and *E*. *coli* kanamycin (50), and DH10β pGEM-derivates ampicillin (100). Cells were either grown as 50-mL cultures in 250-mL flasks, or in 1-mL volumes in 96 deep-well plates.

### Growth curve analysis

To determine the MH96 growth rate over 24 h, 50-mL cultures were sampled each hour for the first 12 h and then every second hour from 12–24 h to measure optical density spectrophotometrically at 600 nm (SmartSpec^TM^Plus, BioRad) and to enumerate colony forming units (CFU) by serial dilution plating on LB agar. To measure CFUs, a dilution series of the culture broth was undertaken using 0.01 M phosphate-buffered saline (PBS, 0.13 8M NaCl, 0.002 7M KCl, pH 7.4, Sigma-Aldrich, Merck, Darmstadt, Germany). Replicate samples at each time point were used and the appropriate dilutions were plated on LB agar. For OD600 > 1, the cell culture was diluted at 1:10 and measured again.

### Exoprotein assessments

In this study the exoproteome is defined as the sum of all proteins in the culture supernatant after centrifugation (2,200 × g in 96-well plates or 5,000 × g of 50-mL cultures for 15 min).

To analyse the exoproteome profile of each of the HESA transposon mutants, the 96 deep-well plates of cell cultures were centrifuged at 2,200 × g (15 min) to seperate the supernatant and cell pellets. The relative size of each centrifuged cell pellet was visually compared with that of strain MH96, and strains showing impaired growth (smaller cell pellet) were omitted from subsequent screens.

To define exoprotein concentrations the supernatant was assessed by Bradford assay using the Protein Assay Kit (BioRad) following the manufacturer’s instructions [30]. A volume of 12 μL of the supernatant was removed from each well and mixed with 220 μL of dye reagent from the Protein Assay Kit in a 96-well microtiter plate and absorbance (595 nm) was measured in a FLUOstar OPTIMA plate reader (BMG LABTECH). Protein concentration was determined by comparing results against a standard curve generated using known concentrations of bovine serum albumin (BSA) (0–500 mg/mL) diluted in LB broth.

Mutants with a similar sized cell pellet but different exoproteome concentration relative to MH96 as determined through modified Bradford assay were further examined by sodium dodecyl sulfate-polyacrylamide gel electrophoresis (SDS-PAGE) using 50-mL cultures.

To assess exoproteins of 50-mL cultures grown for 1h, 1 mL of the culture was transferred into a 1.7 mL microcentrifuge tube and the sample pelleted at 5,000 × g for 15 min. The supernatant was removed and filter-sterilised using a 0.22-μm polyvinylidene fluoride syringe filter (Merck, Darmstadt, Germany). The cell pellet was resuspended in 1 mL of MilliQ water and diluted in 1:10 in water. Supernatants were then used for Bradford assay as described above and for standard SDS-PAGE which was performed as described by Laemmli [31]. Proteins were visualized by silver staining according to Blum et al. [32].

### Transposon mutagenesis

*Escherichia coli* strain ST18 transformed with pKRCPN2 (encoding the miniTn5-derived transposon Tn-DS1028uidAKm) was conjugated to MH96 as outlined by Mesarich *et al*. [33]. The bacteria were then plated on LB agar plates supplemented with 100 μg/mL kanamycin selective for Tn-DS1028uidAKm.

### DNA isolation, manipulation, and sequencing

Standard DNA techniques were performed as described in Sambrook et al. [34]. Genomic DNA was isolated using PrepMan Ultra Sample Preparation Reagent (Thermo Fisher). PCR amplicons were purified using a High Pure PCR Product Purification Kit (Roche). As described by Mesarich et al. [35], the purified genomic DNA, nested primer and a random primer were used in arbitrary PCR. Amplicons were ligated into the pGEM plasmid using the pGEM T-Easy Kit (Promega, Madison, WI). Plasmid DNA was isolated using a High Pure Plasmid Isolation Kit (Roche) and quantified using NanoDrop2000 Spectrometer (ThermoScientific). The inserted sequence was validated with primer M13F (5’ GTAAAACGACGGCCAGT 3’) and M13R (5’ GCGGATAACAATTTCACACAGG 3’) using Macrogen Sequencing Services (Macrogen Inc., Seoul, Republic of Korea).

### Sequence assembly and bioinformatic analysis

DNA sequences were trimmed and aligned against the genome of strain MH96 (GenBank accession number NZ_CP010029.1) using the Map to Reference function of Geneious 10.0.9 [36]. Protein function prediction was performed using the Protein Fold Recognition Server PHYRE2 (http://www.sbg.bio.ic.ac.uk/phyre2/html/page.cgi?id=index). Amino acid sequences in FASTA format were uploaded to the PHYRE2 webpage and run with default settings. For further functional protein predictions, the protein sequence was analysed using BLASTP (https://blast.ncbi.nlm.nih.gov/Blast.cgi) with default settings. The COG categories were assigned using the standalone COG software [37].

### Data analysis

Data collected in the growth curve experiment was visualized using the GraphPad Prism 7.04 software (GraphPad).

## Results

### Defining the point of measurable exoprotein release in MH96

**Fig 1:**
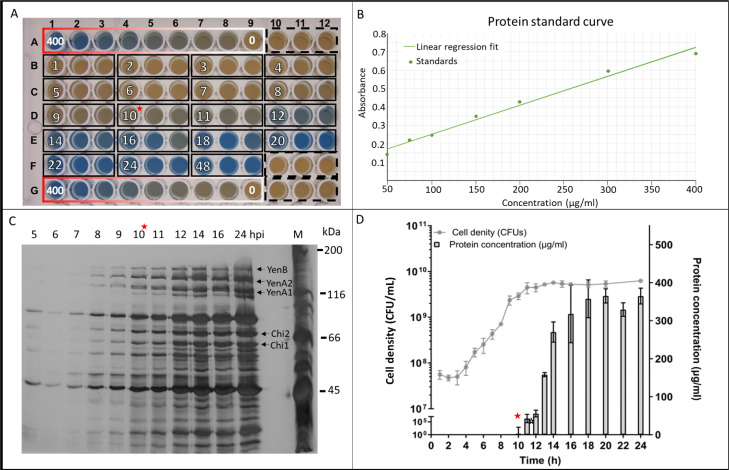
Assessment of exoprotein concentration in a 50-mL culture of MH96. (A) Photograph of a Bradford assay conducted in a 96-well microtiter plate. Wells contained 50 μL of culture supernatant collected at 1–24 hpi and 48 hpi. Each timepoint (white numbers) was measured in triplicate (black solid boxes). The dashed black box indicates uninoculated LB broth blanks. The red-to-white gradient box indicates BSA concentrations (400, 300, 200, 150, 100, 75, 50, 25, and 0 μg/mL) used to generate a standard curve in B. Red star denotes the time point at which exoprotein was detectable (0.2 μg/mL) by Bradford assay. (B) Protein standard curve calculated using the BSA concentrations as indicated in A. (C) Silver stained SDS-PAGE of the culture supernatants assessed at the listed time points. Indicated are protein bands of the Yen-TC (arrows) [25, 38]. M, denotes Bio-Rad broad range marker with molecular weights (kDa). (D) Growth curve and protein secretion of MH96 cultured at 25°C and 200 rpm over 24 h. OD_600_ (●), CFU (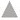), and protein concentration (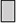), as measured from the 96-well microtiter plate in (A), were assessed at the indicated time points. Protein concentrations were calculated using the standard curve in (B). Data are presented as the mean ± standard deviation. A,B,C) Red asterisk denotes sample where initial exoprotein release was measured.

To understand protein secretion in MH96, we first investigated the correlation between cell growth and exoprotein release. Assessment of the 50-mL cell culture from 1–24 h post-inoculation (hpi) showed an exponential increase from 5.0 × 10^7^ to 7.0 × 10^8^ CFUs between 3–8 h. Cell density peaked at 5.7 × 10^9^ CFU/mL (OD_600,_ 6), at 14 hpi (Fig 1D). Using the Bradford assay to measure protein concentration in the supernatants, initial exoprotein of 42.9 ± 9 μg/mL was detected at 11 hpi (4.4 × 10^9^ CFU/mL) and increased over time, reaching 364.9 ± 20 μg/mL at 24 hpi (5.4 × 10^9^ CFU/mL) (Fig 1A and 1B). Assessment of culture supernatants by SDS-PAGE revealed the presence of exoprotein as early as 5 hpi (Fig 1C). Based on this information, it was determined that the optimal timeframe for collection and analysis of exoprotein content in MH96 LB broth cultures by Bradford assay was at 16 hpi (OD_600_ = 5.5) with exoprotein levels of ~ 317 μg/mL.

In preparation for the HESA, we first validated that the cell density of MH96 50-mL LB broth cultures correlated with cell numbers of cultures grown in 96-well plates at timepoints of exoprotein release. At 16 hpi the MH96 culture incubated in a 96-well plate reached 2.4 × 10^9^ CFU/mL with an exoproteome concentration of 277 μg/mL compared to 5.2 × 10^9^ CFU/mL with exoproteome concentration of 242 μg/mL of 50 mL culture grown in a 250-mL shake flask. The similar cell growth at 16 h between 50-mL cell cultures and 96-deep well culture numbers validates the use of the 96-deep well culture plates in the HESA.

### The high-throughput exoprotein screening assay (HESA)

**Fig 2:**
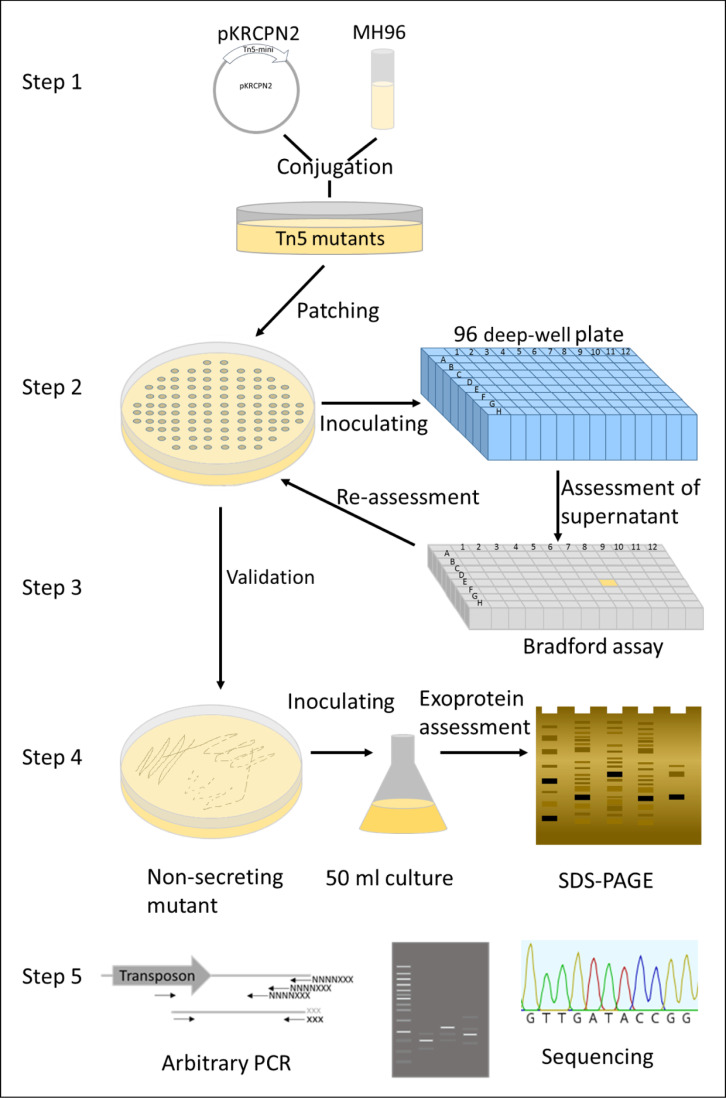
Flowchart of the steps involved in the high-throughput exoproteome screening assay (HESA). **Step 1**: Construction of a transposon mutant library by conjugation of *E*. *coli* pKRCPN2 (Tn*5*-harboring plasmid) with MH96. **Step 2:** Eighty random mutants per transformation event were patched onto a selective LB agar plate. The same tip was used to inoculate a single well of a 96-well plate, which was incubated at 25°C and 200 rpm for 24 h. **Step 3:** Following centrifugation at 2,200 × *g* for 15 min, 12 μL of the supernatant were transferred to a 96-well plate for exoprotein quantification by Bradford assay. Non-secreting mutants were distinguishable by less blue coloration in the assay (indicated as a yellow well). All putative secretion-altered mutants were re-assessed using the protein assay (Steps 2–3) and subsequently stored. **Step 4:** Mutants with an altered secretion profile were then validated through SDS-PAGE assessment of the exoprotein content derived from 50-mL LB broth cultures at 16 hpi. **Step 5:** Transposon insertion points within the genomes of non-secreting mutants were determined by arbitrary PCR and the resultant sequences aligned to the MH96 genome sequence (GenBank accession number NZ_CP010029.1).

To determine genes involved in exoprotein release, the exoproteome of random transposon mutants were assessed in a 5-step screening assay as outlined in Fig 2.

#### Cultivation of MH96 transposon mutants in 96-deep well plates

**Fig 3:**
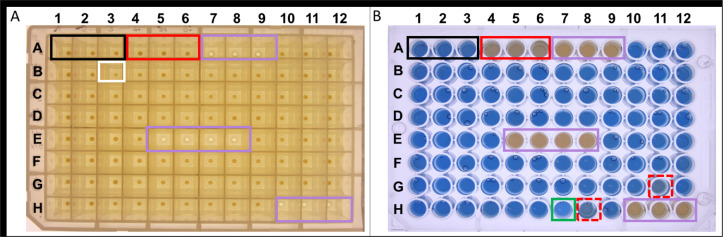
Representative photographs of exoprotein assessment in a 96-well master plate. (A) Inoculation plate post-centrifugation at 16 hpi on a lightbox. Wells A1–3 (black box) contain MH96 (wild-type); A4–6 (red box) contain K18 (non-secretion control); A7–9, E5–8, and H10–12 (purple box) contain LB broth blanks. The pellet sizes of the strains were compared with those of MH96 (black box) and mutant strains with smaller cell pellets were identified (white box). LB-only blanks (purple boxes). (B) Bradford protein assay of supernatants from 96-well master plate shown in panel A. No color change was observed for strain K18 (non-secreting control, red box with solid lines). Transposon mutants with decreased (red boxes with dashed lines) and increased (green box) exoprotein compared to the wild type (black boxes) are indicated.

Eighty mutants resulting from each independent conjugation event (total events = 50) were independently patched onto LB agar plates containing kanamycin, selective for the transposon, and into 1-mL volumes of LB broth with kanamycin in the wells of 96-well deep-well plates (Nunc 442404). Additionally, three wells without antibiotics were inoculated with MH96 and a non-secreting MH96 strain, K18 (S1 Table of S1 File), and 10 wells containing only LB broth were used as blanks (Fig 3). Plates were sealed with semi-permeable seals AeraSeal (cat BS25, ExcelScientific, USA) and incubated for 16 h at 25°C in a Minitron incubator shaking at 200 rpm.

Following incubation, the deep-well plates were checked for contaminants by visually observing the presence of growth in the uninoculated wells. When no growth (no pellet) in the non-inoculated wells (blanks) were observed, the 96 well plates were centrifuged at 2,200 × *g* (15 min), and cell pellets were visually assessed by placing each 96 well plate upright onto a lightbox. Mutants with cell pellets that varied in size (Fig 3A well B3) relative to MH96 (wells A1-A3, Fig 3), were removed from the mutant pool, while mutants with similar cell pellet size compared to MH96 were taken forward for exoprotein assessment of the culture supernatant.

To define the exoprotein concentration, the supernatants of the spun down 96-deep well plate were assessed using Bradford assay. Transposon mutants with decreased exoprotein concentration compared to MH96 were placed through a second iteration of the 96-well-based Bradford assay (steps 2–3, Fig 2).

Through the screening of 4,080 MH96 Tn*5* transposon mutants using the HESA, 50 mutants (H1-H50) were identified with cell pellets similar in size to MH96 and altered levels of exoprotein relative to wild-type strain MH96 and its non-secreting MH96 derivative, strain K18.

#### Assessment of MH96 transposon exoprotein mutant strains

**Fig 4:**
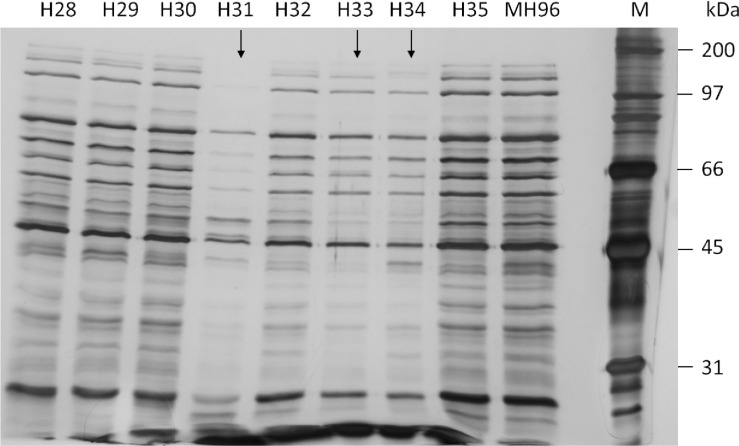
SDS-PAGE (10%) of culture supernatant of representative HESA-derived Tn5 mutants H28–35 (Table 1) cultured for 16 h. Transposon insertion sites, and CFUs compared to MH96 are listed in Table 1. Black arrows denote altered exoproteome profiles with decreased band intensities in transposon mutants relative to MH96. M, Bio-Rad broad-range marker.

To enable assessment of the protein profile by SDS-PAGE, each of the 50 prospective MH96 transposon mutants with altered exoprotein concentration were independently grown in 50-mL cultures at 25°C and 200 rpm shaking for 16 h. The cultures were assessed for CFUs and 1 mL was pelleted by centrifugation (5,000 × g, 15 min) from where 25 μL of supernatant was retained for Bradford assay and SDS-PAGE (Fig 4) and 25 μL of a resuspended and 1:10 diluted cell pellet was assessed by SDS -PAGE (S1 Fig of S1 File).

Mutants in which band intensities or the banding pattern of the supernatant and or the cell pellet deviated from MH96 observed via SDS-PAGE (Fig 4, S1 Fig of S1 File) are of specific interest, as the deviation in the exoproteome suggests a role of the mutated gene in exoprotein release. A decrease in exoprotein concentration measured by Bradford-assay, often coincided with reduction of band intensities of the entire banding profile observed by SDS-PAGE. As an example, the mutant strains H31, H33 and H34 (Fig 4, S1 Fig of S1 File) where H34 exhibited a similar cell growth with 6.4 × 10^9^ CFU/mL as the wildtype MH96 (5.2 × 10^9^ CFU/mL), while the H31 and H33 mutants had slightly lower respective cell densities of 2.3 × 10^9^ CFU/mL and 2.9 × 10^9^ CFU/mL (Table 1). Based on cell growth and the similar observed band intensity of the associated cell pellets (S1 Fig of S1 File), the H31 and H33 mutants are considered valid exoprotein mutants.

**Table 1:** MH96 HESA transposon mutants generated in this study, grouped by COG category.

Mutant	Locus-Tag	Gene description	COG*	COG_description	Gene length (bp)	Insertion in gene/*intergenic* (bp)	Exoproteome (BA^†^) compared to MH96	FC^#^ CFU (log10) compared to MH96 (%^‡^)
H15	PL78_01430	Type II citrate synthase/*gltA*	C	Citrate synthase	1284	1242	-52%	0.93 (-77%)
H33	PL78_01430	Type II citrate synthase/*gltA*	C	Citrate synthase	1284	793	-42%	0.97 (-47%)
H55	PL78_09650	Dihydrolipoyl dehydrogenase/*lpdA*	C	Pyruvate/2-oxoglutarate dehydrogenase complex, dihydrolipoamide dehydrogenase (E3) component or related enzyme	1326	1250	-34%	0.99 (-26%)
H11	PL78_12985	Malate dehydrogenase/*mdh*	C	Malate/lactate dehydrogenase	939	205	-89%	0.93 (-80%)
H08	PL78_08330	Aspartate ammonia lyase/*aspA*	E	Aspartate ammonia-lyase	1437	149	-76%	0.97 (-43%)
H16	PL78_09380	Arginine decarboxylase	E	Arginine decarboxylase (spermidine biosynthesis)	1980	707	-67%	0.99 (-25%)
H42	PL78_07560	Guanosine polyphosphate pyrophosphohydrolase/*ppx*	F	Exopolyphosphatase/pppGpp phosphohydrolase	1494	877	-12%	1.02 (+60%)
H48	PL78_00755	Phosphoglucosamine mutase/*glmM*	G	Phosphomannomutase	1371	145	-15%	0.98 (-31%)
H24	PL78_03735-PL78_03740	N.A. ^§^	K	Intergenic (Yen7 regulator—Chitinase)	N.A.	193 5′ y*en7*	+36%	0.97 (-51%)
H34	PL78_04800-PL78_04805	N.A.	K	Intergenic (DNA-binding protein H-NS—Thymidine kinase)	N.A.	159 5′ *hns*	-50%	1.01 (+19%)
H56	PL78_04800-PL78_04805	N.A.	K	Intergenic (DNA-binding protein H-NS—Thymidine kinase)	N.A.	357 5′ *hns*	-11%	0.97 (-49%)
H19	PL78_11480-PL78_11485	N.A.	K	Intergenic (peroxiredoxin—global nitrogen regulator NtcA)	N.A.	153 5′ *ntcA*	-74%	1.00 (+3%)
H29	PL78_14480-PL78_14485	N.A.	K	Intergenic (DNA mismatch repair ATPase MutS—YscW T3SS lipoprotein chaperone)	N.A.	160 3′ *mutS*	-46%	1.01 (+35%)
H25	PL78_15400	Hypothetical protein	K	DNA-binding transcriptional regulator, GntR family	900	581	-59%	1.02 (+43%)
H30	PL78_15400	Hypothetical protein	K	DNA-binding transcriptional regulator, GntR family	900	767	-54%	1.04 (+132%)
H04	PL78_17385-PL78_7390	N.A.	K	Intergenic (phoB-like regulator—holin)	N.A.	120 5′ *holA*	-97%	0.94 (-74%)
H31	PL78_17385-PL78_7390	N.A.	K	Intergenic	N.A.	237 5′ *holA*	-92%	0.96 (-58%)
H46	PL78_17385-PL78_7390	N.A.	K	Intergenic	N.A.	70 5′ *holA*	-91%	0.98 (-30%)
H12	PL78_17385	Hypothetical protein	K	HTH-motif transcriptional regulator	419	139	-98%	1.03 (+113%)
H02	PL78_07555	DNA helicase/*recQ*	L	Superfamily II DNA and RNA helicase	1290	1100	-68%	1.00 (+4%)
H20	PL78_07980	LPS heptosyltransferase/*waaC*	M	ADP-heptose:LPS heptosyltransferase	966	791	-91%	0.92 (-83%)
H05	PL78_00700	LPS paratose synthase	M	Nucleoside-diphosphate-sugar epimerase	867	256	-78%	0.96 (-58%)
H03	PL78_00710	LPS/*murJ*	M	Membrane protein involved in the export of O-antigen and teichoic acid	1425	554	-68%	0.98 (-29%)
H07	PL78_00710	LPS/*murJ*	M	Membrane protein involved in the export of O-antigen and teichoic acid	1425	774	-83%	0.91 (-86%)
H09	PL78_00710	LPS/*murJ*	M	Membrane protein involved in the export of O-antigen and teichoic acid	1425	252	-78%	0.89 (-91%)
H18	PL78_00725	LPS/*glgA*	M	Cell membrane biogenesis / mannosyltransferase	1016	266	-25%	0.93 (-80%)
H13	PL78_07500	LPS O-Antigen translocase	M	membrane protein involved in acid resistance	1266	114	-80%	0.96 (-57%)
H21	PL78_07505	LPS Aminotransferase/*wecE*	M	dTDP-4-amino-4,6- dideoxygalactose transaminase	1131	816	-49%	1.01 (+26%)
H22	PL78_07505	LPS Aminotransferase/*wecE*	M	dTDP-4-amino-4,6- dideoxygalactose transaminase	1131	791	-43%	1.02 (+46%)
H32	PL78_11175	Potassium transporter/*trkA*	P	Trk K^+^ transport system, NAD- binding component	1377	469	-70%	0.99 (-22%)
H27	PL78_04165	non-ribosomal peptide synthetase	Q	Thioesterase domain of type I polyketide synthase or non-ribosomal peptide synthetase	5535	4419	-59%	1.01 (+21%)
H53	PL78_04270	Hypothetical protein	T	Regulator of *sirC* expression, contains transglutaminase-like and tetratricopeptide repeat domains	810	795	-16%	0.98 (-31%)
H23	PL78_03850	QS/N-acyl-L-homoserine lactone synthase	T	N-acyl-L-homoserine lactone synthetase	651	450	-51%	1.03 (+86%)
H45	PL78_03850	QS/N-acyl-L-homoserine lactone synthase	T	N-acyl-L-homoserine lactone synthetase	651	163	-40%	1.02 (+47%)
H41	PL78_11650	intracellular growth attenuator protein/IgaA	S	intracellular growth attenuator protein	2151	2029	-14%	0.99 (-17%)
^¶^K18	N.A.	N.A.	N.A.	N.A.	N.A.	N.A.	-98%	1.05 (+194%)

* COG (Clusters of Orthologous Groups) classifications retrieved from Hurst et al. [26]: C, energy production and conservation (11%); E, amino acid metabolism and transport (5.5%); F, nucleotide metabolism and transport (2.8%); G, carbohydrate metabolism and transport (2.8%); K, transcription (33.3%); L, replication and repair (2.8%); M, cell wall/membrane/envelope biogenesis (22.2%); P, inorganic ion transport and secretion (2.8%); Q, secondary structure (2.8%); T, signal transduction mechanisms (11%); S, unknown function (2.8%).

† BA, Bradford assay at 16 hpi: Exoprotein concentrations of all samples were calculated by standard curve y = 0.001661 * x + 0.1834, with x = measured absorption and compared to wild-type strain MH96 exoprotein concentration of 277 μg/mL.

‡ CFU difference of mutants to MH96 based on 50-mL cultures. % values are MH96 (defined as 100% growth) ± difference of the mutant divided by MH96.

# FC, Fold change calculated by dividing MH96 log10CFU by mutant log10CFU.

§ N.A., not applicable, insertion located in the intergenic region.

¶ *Yersinia entomophaga* strain K18, used as the non-secreting control, refer to S1 Table of S1 File for strain details.

Using the HESA approach, 34 of the 50 mutants were identified with exoprotein concentration reduction by >25% and one mutant H24, with elevated exoprotein concentration of 36% relative to MH96 (Table 1). All 35 mutants have similar CFU values, not less than 5.2 × 10^8^ CFU/mL (<0.1 log10 FC), compared to MH96. Of the 35 mutants, 25 had CFU values above 2.6 × 10^9^ CFU/mL (<0.05 log10 FC) compared to MH96.

#### Characterization of MH96 Tn5 insertion sites in mutants with altered exoprotein profile

Through arbitrary PCR and DNA sequencing, the transposon insertion sites of each of the mutants were identified (Table 1). Based on predicted function and Clusters of Orthologous Groups (COG) assignments of the mutants with decreased exoprotein concentrations, 11 insertions were found in transcriptional regulators and intergenic regions (S2 Fig of S1 File). These regions included: i) the intergenic region of PL78_14480, encoding for DNA mismatch repair protein MutS, and PL78_14485; ii) the intergenic region of PL78_17385, a putative PhoB-like regulator and PL78_17390, encoding a phage-like holin; iii) the locus PL78_04800, encoding a histone-like nucleoid-structuring protein (H-NS); and iv) the locus PL78_14485 encoding a hypothetical protein located in the T3SSY2 cluster (S2 Fig and S2 Table of S1 File). Other transposon insertion points were identified in genes involved in metabolism, cell wall biogenesis, lipopolysaccharide (LPS) synthesis and quorum sensing (QS) (Table 1). Some gene clusters were identified, where multiple independent transposon insertions were found (S3 Fig of S1 File).

One hyper-secreting strain was identified in the HESA (H24) (S1 and S4 Figs of S1 File). The transposon insertion of H24 was located in the intergenic region between *yen7*, a hypothetical HTH-type transcriptional regulator, and *chi1* of the Yen-Tc gene cluster.

#### Assessments of MH96 genes with multiple transposon insertion points

**Fig 5:**
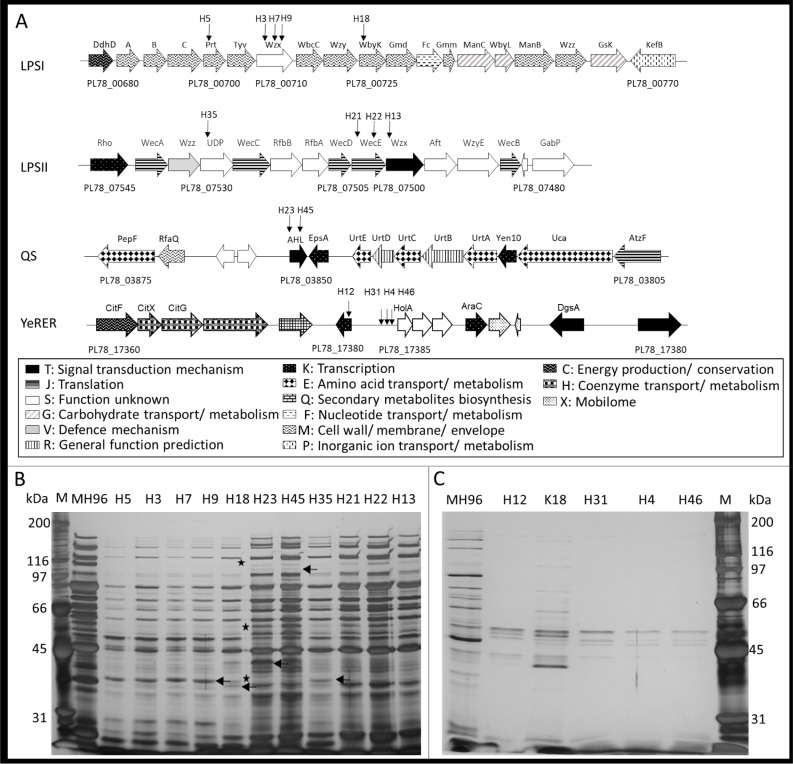
Transposon insertion mutants. (A) Schematic depicting HESA-selected transposon insertion within lipopolysaccharide clusters LPSI, LPSII, the quorum sensing cluster and the YeRER. Vertical arrows denote transposon insertion points. Patterned horizontal arrows reflect COG classifications as indicated in boxes. (B) SDS-PAGE of culture supernatant from 16-h cultures of the strains indicated in A of the cluster LPSI: H5, H3, H7, H9, H18; LPSII: H13, H21, H22, H35 and QS H23, H45. Arrow (←) and asterisk (*) denotes bands that increased or decreased in intensity, respectively, relative to MH96. M, Bio-Rad broad-range marker. (C) SDS-PAGE of the culture supernatant of transposon mutants located within (H12) PL78_17385 and 5’ of PL78_17390 (H4, H31 and H46). The MH96 wildtype and its non-secreting derivative K18 are indicated. M, Bio-Rad broad-range marker. Refer to Table 1 for associated transposon insertion sites, exoprotein release and CFU information.

Five transposon insertions were co-located in the LPSI cluster (Fig 5A and Table 1), required for O-antigen synthesis [28]. Of these, three independent insertions were in genes coding for an inner membrane protein involved in murein biosynthesis (Wzx), with the remaining two insertions in paratose synthase (*prt*) and mannosyltransferase (*wbyK*) genes. Four additional transposon insertions were identified in the LPSII cluster, which catalyzes the biosynthesis of an enterobacterial common antigen [29]. These insertions were in genes encoding a uridine-5ʹ-diphosphate (UDP) epimerase, an aminotransferase (WecE), and the O-antigen translocase (Wzx) (Fig 5A and Table 1). SDS-PAGE assessment of these mutants indicated that alterations in exoprotein release were similar between the various mutations within LPSII cluster and MH96 whereas mutations in the LPSI cluster (H3, H5, H7, H9 and H18) led to decreased exoprotein release (Fig 5B).

Of significance are the three insertions in H4, H31 and H46 within the intergenic region of PL78_17385, encoding for a putative transcriptional regulator, and within the ORF PL78_17390–17400, encoding for a holin, endopeptidase and putative spanin-protein, as well as the transposon insertion in PL78_17385 in H12 (Fig 5). The exoprotein profiles of those mutants showed a reduction in the exoprotein with only a few visible bands, which was also observed in the non-secreting strain K18. In comparison to MH96 that released 277 ug/mL, K18 released 5 ug/mL, and the HESA mutants H12, H4, H31 and H46 released 6 μg/mL, 9 μg/mL, 21 μg/mL and 25 μg/mL, respectively. The strains K18 (1.6 × 10^10^ CFU/mL) and H12 (1.2 × 10^10^ CFU/mL) showed an increase in CFUs compared to MH96 (5.2 × 10^9^ CFU/mL), while strains H46 (3.8 × 10^9^ CFU/mL), H4 (1.4 × 10^9^ CFU/mL) and H31 (2.3 × 10^9^ CFU/mL) showed lowered CFUs compared to MH96 (Table 1). Due to the almost complete abolishment of exoproteins resulting from the mutations within the PL78_17385–17400 region, the cluster was designated as the *Yersinia* region of exoprotein release (YeRER).

## Discussion

To identify genes involved in MH96 exoprotein production, a high-throughput screening method, HESA, was developed and used to screen transposon mutants for alterations in the exoprotein profile. Using the HESA, 35 mutants with altered exoprotein concentrations relative to wild-type strain MH96 were identified. Several transposon insertions were located in the same gene clusters (e.g. LPSI & LPSII), revealing these clusters as essential to MH96 exoprotein release. In line with our goal of defining genes involved in exoprotein production and release, transposon insertions in AHL synthetase, four insertions in transcriptional regulators, and eight insertions located in intergenic regions adjacent to transcriptional regulators, including H-NS and a PhoB-like regulator, were identified. The identification of independent insertions in the same gene or gene clusters validates sufficient mutants were made for this strain and demonstrates the effectiveness of the HESA in the identification of genes involved in exoprotein release.

Transposon insertions in the LPSI and LPSII operons resulted in reduced levels of exoprotein. Interestingly, LPSII mutants with insertions in *wecE*, encoding dTDP-4-amino-4,6-dideoxygalactose transaminase, not only showed reduced exoprotein concentration but also higher cell densities relative to MH96. In *Pseudomonas aeruginosa* alteration of the LPS resulted in the decreased secretion of elastase LasB and the lipase LipA [39]. The secretion of hemolsyin in *E*. *coli* and proteases in *Erwinia chrysanthemi was reduced in LPS mutants [40].* Crhanova *et al*. [41] determined that mutation of LPS synthesis gene *rfaC*, (LPS heptosyltransferase), in *Salmonella enterica* serovar Typhimurium strain LT2 abolished protein secretion. The authors suggested that structural changes in the LPS core interfered with the assembly of membrane-bound machinery such as the T3SS and flagella. It is plausible that a similar, yet to be defined, mechanism is present in MH96, leading to a decrease in exoprotein production, although further study is required.

Further transposon insertions were identified in the MH96 AHL region, which is required for QS. In *Y*. *pseudotuberculosis*, QS modulates T3SS and the Yop effector, which is encoded on the pYV plasmid specific to the human pathogens *Y*. *enterocolitica*, *Y*. *pestis*, and *Y*. *pseudotuberculosis* [42]. In *Serratia liquefaciens* QS regulates T1SS [43] while in *P*. *aeruginosa*, QS controls Type 2, 3, and 6 secretion systems, revealing the importance of QS in protein secretion [44–46]. Despite the decrease in exoprotein release in the QS mutant H23 further studies are needed to validate a direct link between QS and the regulation of Type 1-6SSs. However, none of the Type 1-6SS genes were identified using the HESA, indicating a low impact of exoprotein release of a single secretion system in the context of the entire exoproteome. Furthermore, Type 3, 4 and 6 secretion systems are typically expressed in the presence of a host cell or other bacteria and are used for host invasion, adherence, or defence against host and other bacteria cells [1, 47, 48], a scenario which does not occur under the *in vitro* conditions used in this study. Of interest was the identification of the H24 insertion located in the intergenic region of the putative transcriptional regulator *yen*7 and *chi*1 of the Yen-Tc operon (S3 Fig of S1 File) that resulted in a hyper exoprotein phenotype observable by SDS PAGE (S4 Fig of S1 File). Though the H24 insertion is located 5’of *chi1* no observable difference in Yen-Tc concentration was observed within the H24 exoprotein profile, signifying this insertion has a global effect on exoprotein production.

Identifying known pathways that are linked to exoprotein release confirms the use of the HESA method which we used in this study to identify genes that are implicated in exoprotein release. Based on the results of this study, we propose that the HESA method applies to any exoprotein-producing bacterial species that is conducive to transposon mutagenesis. To enable this, a growth medium in which a high level of exoproteome production is achieved should be defined by analyzing the exoproteome profile by SDS-PAGE. At present, 0.2 μg of BSA/mL is detectable by plate reader and a concentration of ≥1 μg/mL is discernible by eye. Regarding the detection of exoproteins in the cell culture and its visual assessment by the Bradford assay, the protein concentration of the crude MH96 supernatant is close to the maximum visual threshold and therefore the assay would be unlikely to detect hyper-secreting strains. In this study, after screening 4000 mutants only one was detected with an increased exoprotein concentration. However, the HESA method could be modified to increase the detection of hyper-secreting strains through adjustment of the threshold by appropriate dilution of the supernatant.

Transport of protein complexes across cell walls is essential to bacterial survival, with protein secretion involved in a variety of functions, including nutrient acquisition, antimicrobial resistance, and the delivery of toxins or other virulence factors by pathogens, enabling the bacteria to attach to and/or invade host tissues [49–51]. The use of HESA will enable the elucidation of the genes underpinning exoprotein production in these and other organisms, from where through reporter studies [52, 53] the environmental cues of exoprotein production can then be determined. In this respect, further research is needed to define the roles of the YeRER, QS and others by HESA-identified gene clusters in MH96 exoprotein production and release.

## Supporting information

S1 Raw images (PDF)

S1 File Contains all the supporting tables and figures. (PDF)
