# Supplementary material for: Identification of genes involved in exoprotein release using a high-throughput exoproteome screening assay in Yersinia entomophaga
Source: PLoS One. 2022 Jan 25;17(1):e0263019. doi: 10.1371/journal.pone.0263019 (PMC8789137; doi:10.1371/journal.pone.0263019)
Supplement: S1 Raw images — (PDF) [file pone.0263019.s001.pdf]

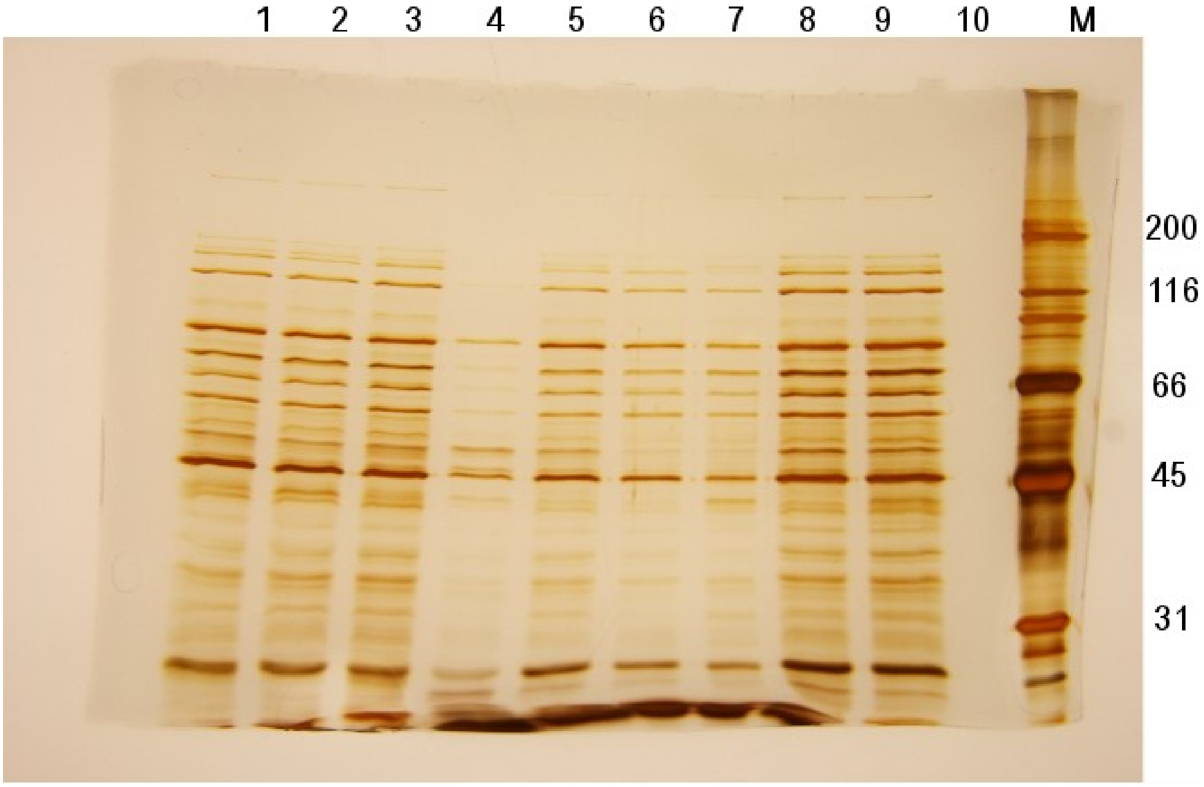

S1_raw_image for Fig 1C. Experimental samples: *Yersinia entomophaga* MH96 culture supernatant of 50-ml culture flask incubated at 25°C with shaking at 200 rpm for 24 h. Equal amount of culture supernatant (15 µL) were loaded per time point. SDS-PAGE was run at 200V for 45 min and visualised by silver stain. Image captured with digital camera Finepix S200EXR (Fujifilm).

Sample order:

1: MH96 culture supernatant of cell culture at 5 hours post induction (hpi)

2: MH96 culture supernatant of cell culture at 6 hours post induction (hpi)

3: MH96 culture supernatant of cell culture at 7 hours post induction (hpi)

4: MH96 culture supernatant of cell culture at 8 hours post induction (hpi)

5: MH96 culture supernatant of cell culture at 9 hours post induction (hpi)

6: MH96 culture supernatant of cell culture at 10 hours post induction (hpi)

7: MH96 culture supernatant of cell culture at 11 hours post induction (hpi)

8: MH96 culture supernatant of cell culture at 12 hours post induction (hpi)

9: MH96 culture supernatant of cell culture at 14 hours post induction (hpi)

10: MH96 culture supernatant of cell culture at 16 hours post induction (hpi)

11: MH96 culture supernatant of cell culture at 24 hours post induction (hpi)

M: Bio-Rad broad range marker (10 µL)

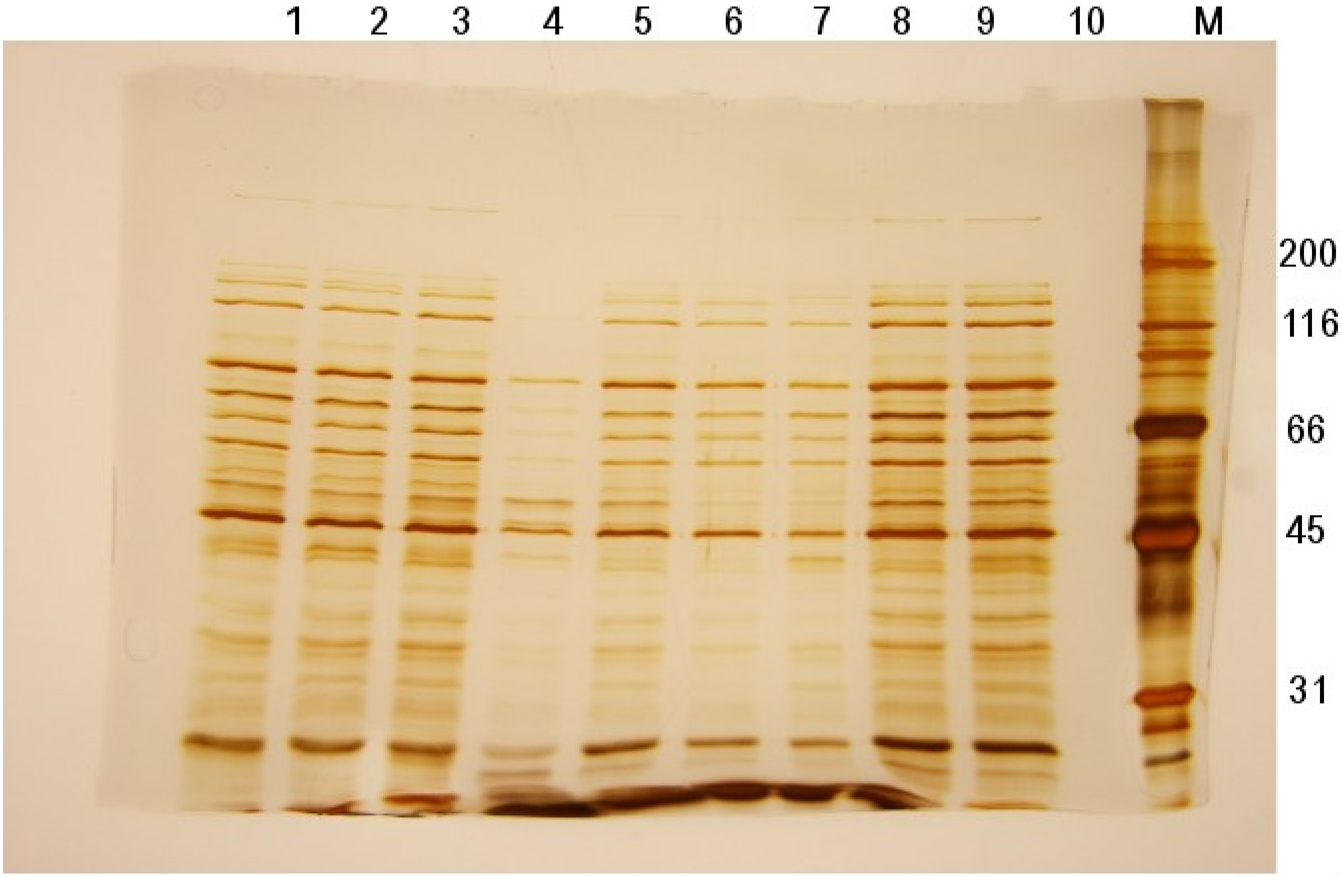

S2_raw_image for Fig 4. Experimental samples: Collected and filter sterilised culture supernatant of Yersinia entomophaga MH96 transposon mutants grown in 50-ml culture flask incubated at 25°C with shaking at 200 rpm for 16 h. Equal amount of culture supernatant (15 µL) were loaded. SDS-PAGE was run at 200V for 45 min and visualised by silver stain. Image captured with digital camera Finepix S200EXR (Fujifilm).

Sample order: 1: MH96 transposon mutant H28

2: MH96 transposon mutant H29

3: MH96 transposon mutant H30

4: MH96 transposon mutant H31

5: MH96 transposon mutant H32

6: MH96 transposon mutant H33

7: MH96 transposon mutant H34

8: MH96 transposon mutant H35

9: MH96

10: empty lane

M: Bio-Rad broad range marker (10 µL)

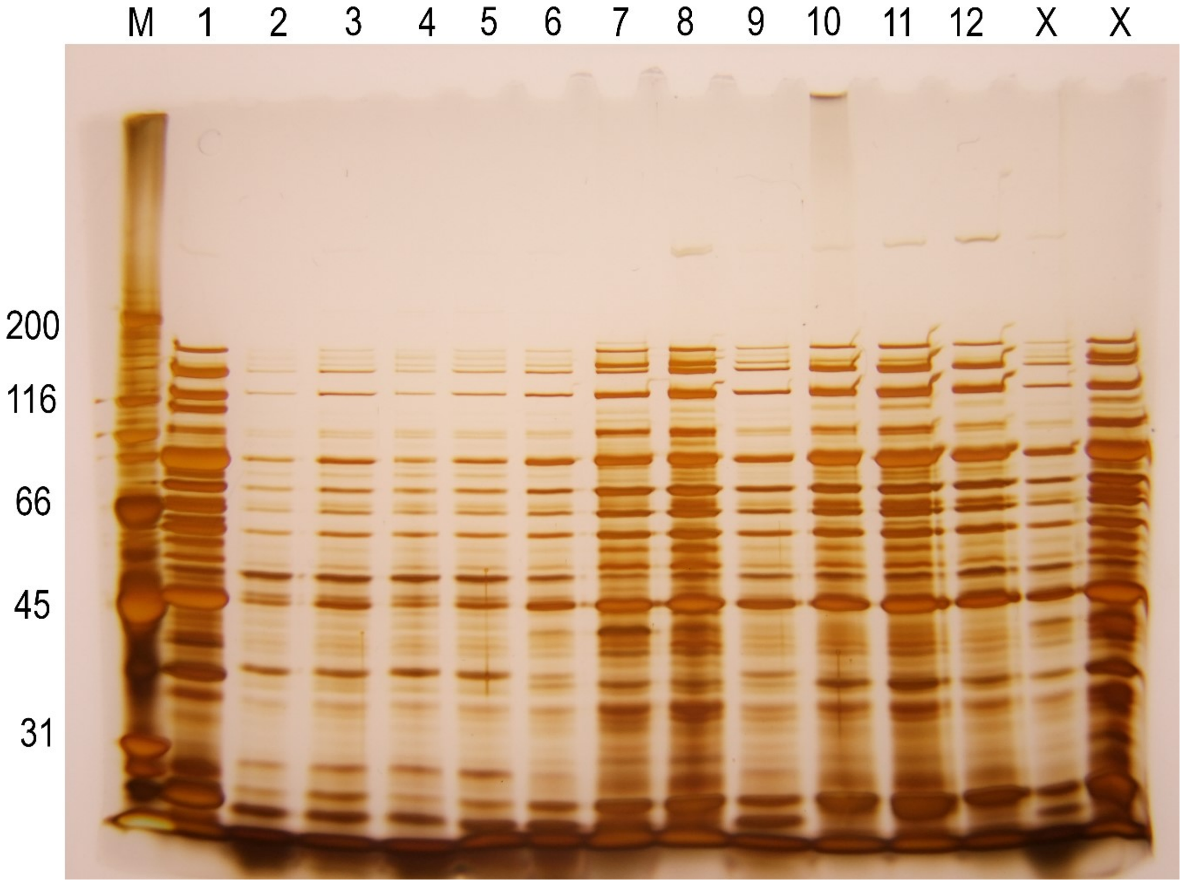

S3_raw_image for Fig 5B. Experimental samples: Collected and filter sterilised culture supernatant of *Yersinia entomophaga* MH96 transposon mutants grown in 50-ml culture flask incubated at 25°C with shaking at 200 rpm for 16 h. Equal amount of culture supernatant (15 µL) were loaded. SDS-PAGE was run at 200V for 45 min and visualised by silver stain. Image captured with digital camera Finepix S200EXR (Fujifilm).

Sample order: M: Bio-Rad broad range marker (10 µL)

1: MH96

2: MH96 transposon mutant H5

3: MH96 transposon mutant H3

4: MH96 transposon mutant H7

5: MH96 transposon mutant H9

6: MH96 transposon mutant H18

7: MH96 transposon mutant H23

8: MH96 transposon mutant H45

9: MH96 ranspson mutant H35

10: MH96 transposon mutant H21

11: MH96 ranspson mutant H22

12: MH96 transposon mutant H13

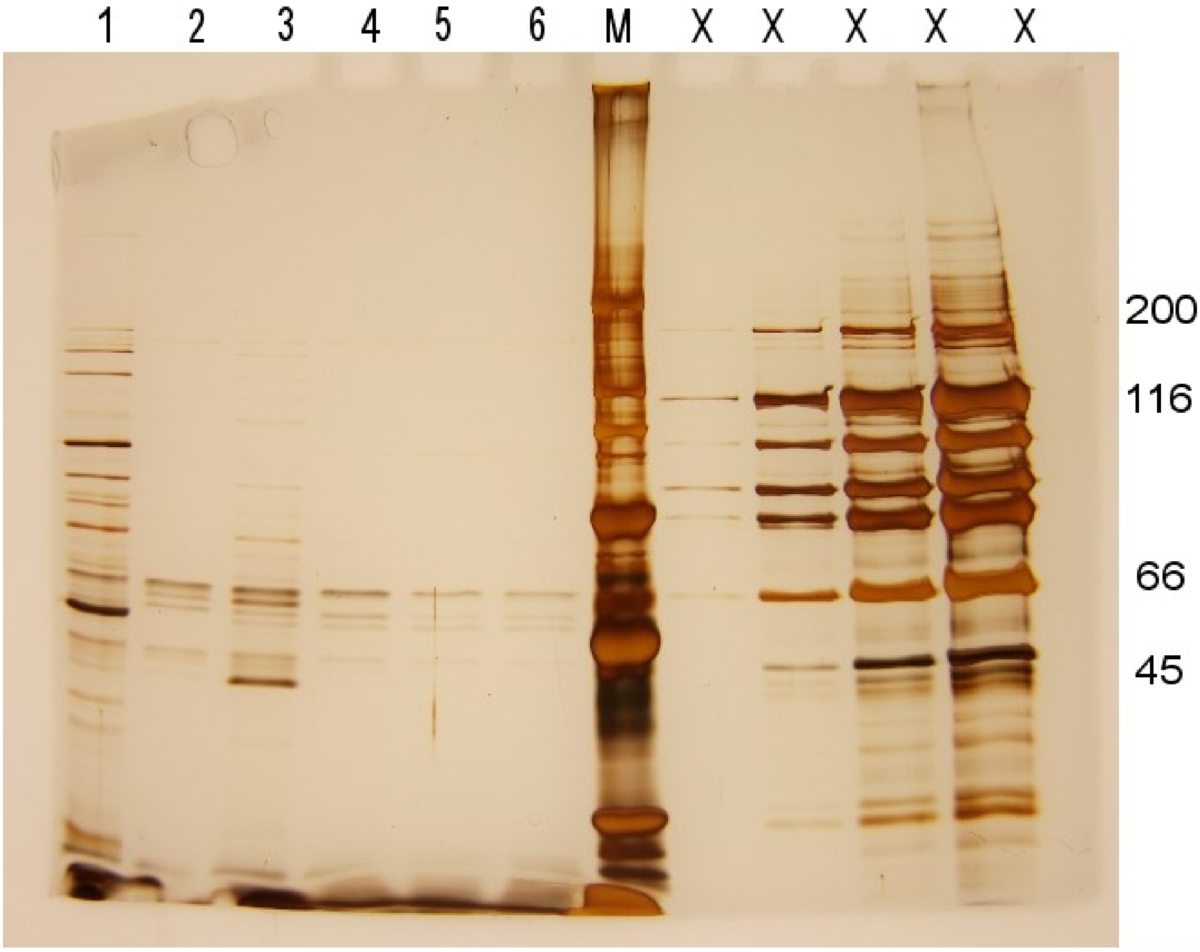

S4_raw_image for Fig 5C. Experimental samples: Collected and filter sterilised culture supernatant of *Yersinia entomophaga* MH96 transposon mutants grown in 50-ml culture flask incubated at 25°C with shaking at 200 rpm for 16 h. Equal amount of culture supernatant (15 µL) were loaded. SDS-PAGE was run at 200V for 45 min and visualised by silver stain. Image captured with digital camera Finepix S200EXR (Fujifilm).

Sample order: 1: MH96

2: MH96 transposon mutant H12

3: MH96 transposon mutant K18

4: MH96 transposon mutant H31

5: MH96 transposon mutant H4

6: MH96 transposon mutant H46

M: Bio-Rad broad range marker (10 µL)

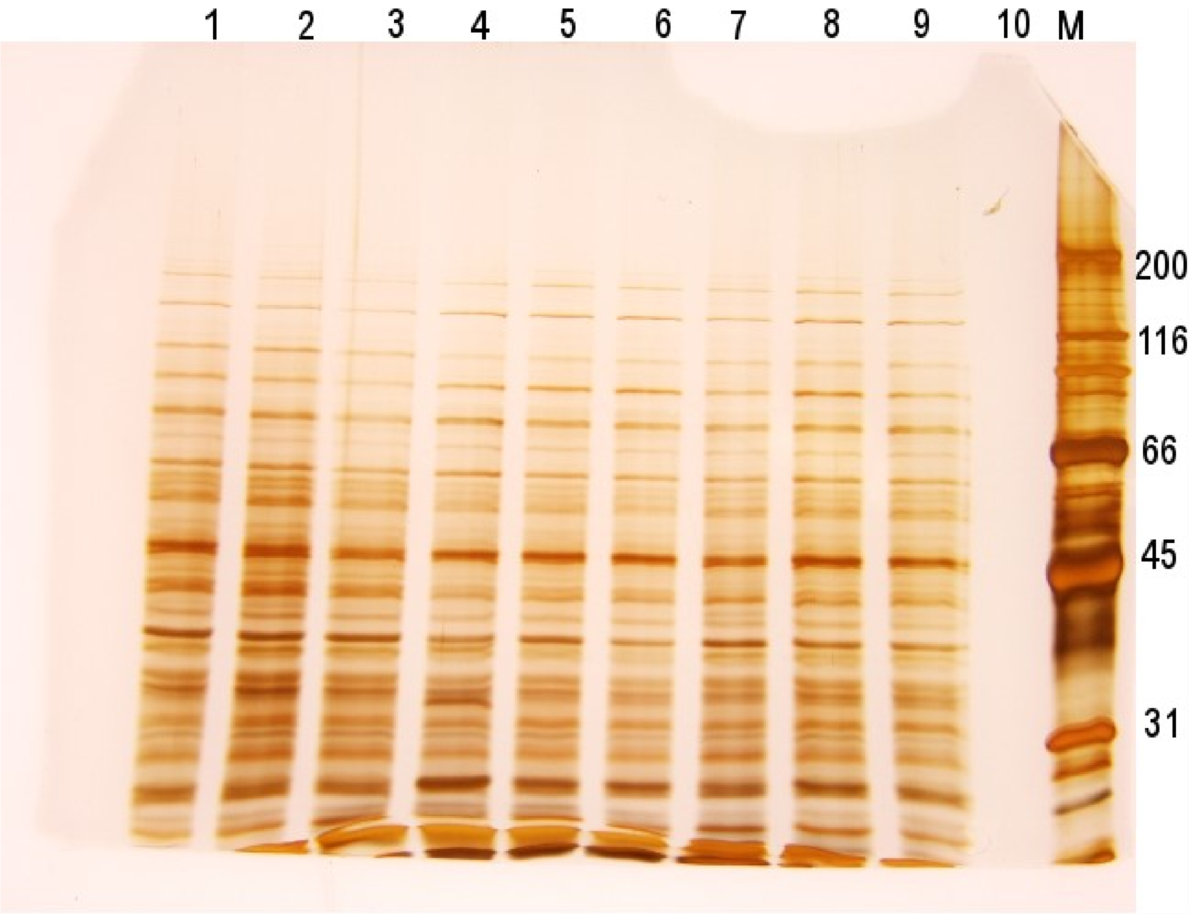

S5_raw_image for Fig S1. Experimental samples: Collected and in water resuspended cell pellets of *Yersinia entomophaga* MH96 transposon mutants grown in 50-ml culture flask incubated at 25°C with shaking at 200 rpm for 16 h. Equal amount of cell pellets (10 µL) were loaded. SDS-PAGE was run at 200V for 45 min and visualised by silver stain. Image captured with digital camera Finepix S200EXR (Fujifilm).

Sample order: 1: MH96 transposon mutant H28

2: MH96 transposon mutant H29

3: MH96 transposon mutant H30

4: MH96 transposon mutant H31

5: MH96 transposon mutant H32

6: MH96 transposon mutant H33

7: MH96 transposon mutant H34

8: MH96 transposon mutant H35

9: MH96

10: empty lane

M: Bio-Rad broad range marker (10 µL)

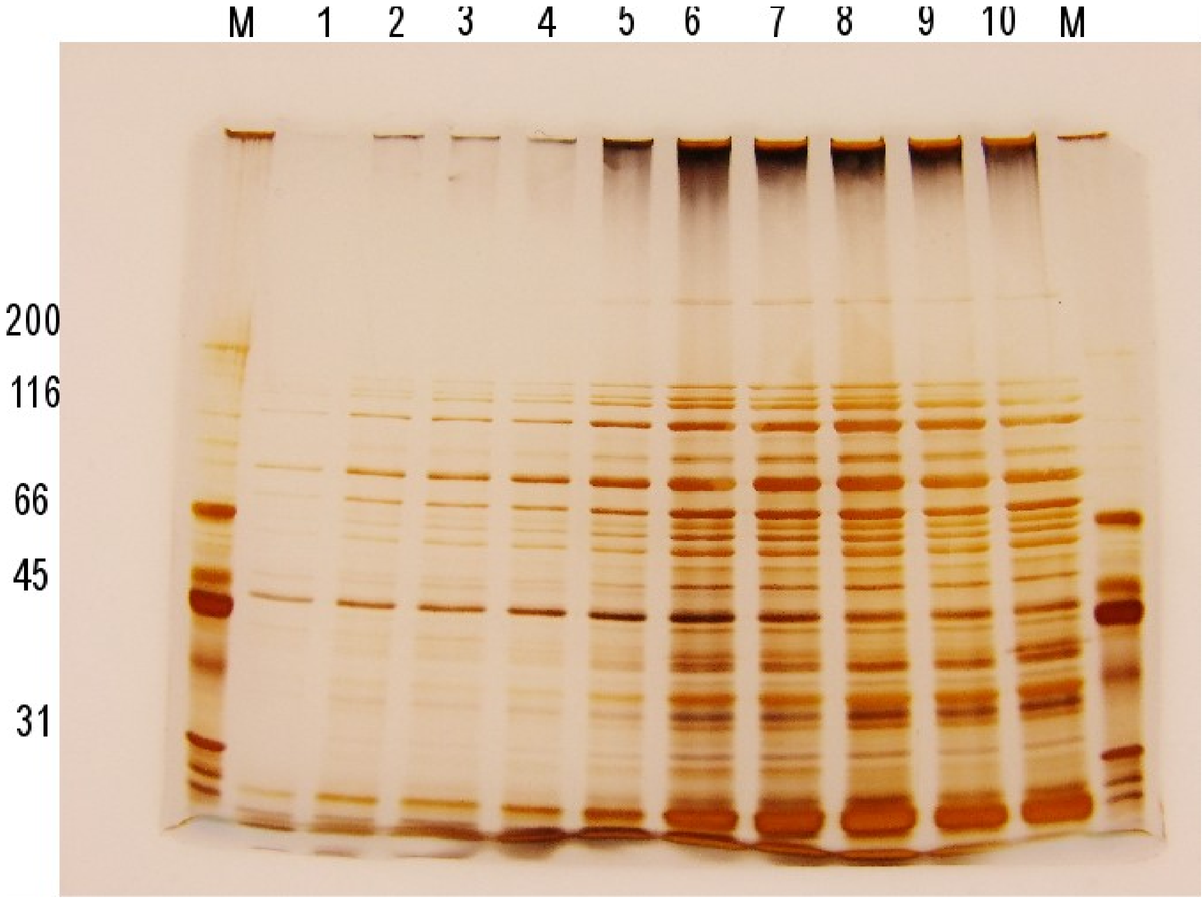

S6_raw_image for Fig S4. Experimental samples: Collected and filter sterilised culture supernatant of *Yersinia entomophaga* MH96 transposon mutants H24 vs. MH96 grown in 50-ml culture flask incubated at 25°C with shaking at 200 rpm over 24h. Samples were taken between 14 h and 24 h post incubation (hpi). Equal amount of culture supernatant (15 µL) were loaded. SDS-PAGE was run at 200V for 45 min and visualised by silver stain. Image captured with digital camera Finepix S200EXR (Fujifilm).

Sample order: 1: MH96 at 14 hpi

2: MH96 transposon mutant H24 at 14 hpi

3: MH96 at 16 hpi

4: MH96 transposon mutant H24 at 16 hpi

5: MH96 at 18 hpi

6: MH96 transposon mutant H24 at 18 hpi

7: MH96 at 20 hpi

8: MH96 transposon mutant H24 at 20 hpi

9: MH96 at 24 hpi

10: MH96 transposon mutant H24 at 24 hpi

M: Bio-Rad broad range marker (10 µL)
