# Supplementary material for: Identification of genes involved in exoprotein release using a high-throughput exoproteome screening assay in Yersinia entomophaga
Source: PLoS One. 2022 Jan 25;17(1):e0263019. doi: 10.1371/journal.pone.0263019 (PMC8789137; doi:10.1371/journal.pone.0263019)
Supplement: S1 File — (PDF) [file pone.0263019.s002.pdf]

S1 Table. Strains and plasmids used in the study.

Strains	Description	Reference
E. coli strains		
ST18	*Escherichia coli* S17 λpir Δ*hemA*	[1]
DH10β	F- *mcrA* Δ(*mrr-hsdRMS-mcrBC*) Φ80d*lacZ*ΔM15 Δ*lacX*74 *endA1 recA1 deoR* Δ(*ara,leu*)7697 *araD*139 *galU galK nupG rpsL* λ-	[2]
Y. entomophaga strains		
MH96	Wild-type strain, isolated from diseased *Costelytra giveni* larvae	[3]
K18	genomic BOX-PCR DNA fingerprint validated spontaneous MH96 non-secreting derivative, isolated at 4 weeks post field trial application of MH96	AgResearch culture collection
H4-53	Transposon mutants of MH96 generated by random Tn5 insertion in this study, Kan^R	This study
**Plasmid**	**Description**	**Source or reference**
pGEM T-Easy	Amp^R, cloning vector, LacZ multi-cloning site	Promega Ltd.
pKRPCN	mini-Tn5-based transposon Tn-DS1028uidAKm, uidA β-glucuronidase (GUS) reporter, tetracycline (Tc) resistance cassette and R6Kγ origin	[4]

S1 References

1. Thoma S, Schobert M. An improved *Escherichia coli* donor strain for diparental mating. FEMS Microbiol Lett. 2009;294(2):127-32. Epub 2009/05/12. doi: 10.1111/j.1574-6968.2009.01556.x. PubMed PMID: 19431232.
2. Lorow D, Jessee J. Max efficiency DH10B: a host for cloning methylated DNA. Focus 12:19. 1990.
3. Hurst MR, Becher SA, Young SD, Nelson TL, Glare TR. *Yersinia entomophaga* sp. nov., isolated from the New Zealand grass grub *Costelytra zealandica*. Int J Syst Evol Microbiol. 2011;61(Pt 4):844-9. Epub 2010/05/25. doi: 10.1099/ijs.0.024406-0. PubMed PMID: 20495033.
4. Mesarich CH, Rees-George J, Gardner PP, Ghomi FA, Gerth ML, Andersen MT, et al. Transposon insertion libraries for the characterization of mutants from the kiwifruit pathogen *Pseudomonas syringae* pv. actinidiae. PloS One. 2017;12(3):e0172790. Epub 2017/03/02. doi: 10.1371/journal.pone.0172790. PubMed PMID: 28249011; PubMed Central PMCID: PMCPMC5332098.

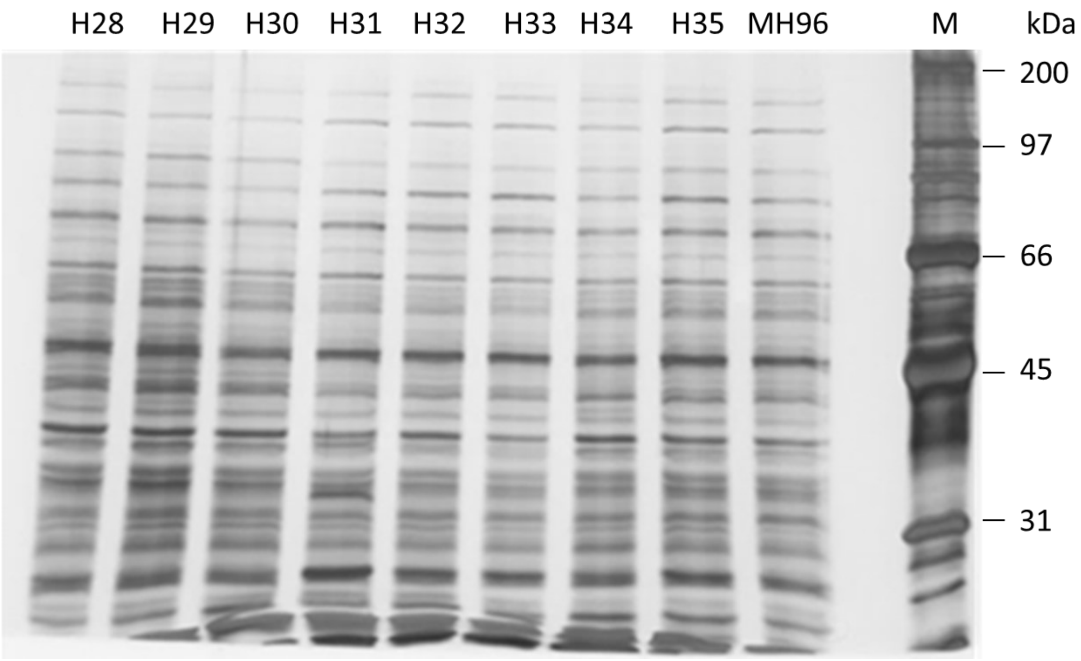

S1 Fig. SDS-PAGE (10%) of 1:10 in sterile MilliQ water diluted cell pellets of representative HESA-derived MH96 Tn5 mutants H28–35 (Table 1) cultured for 16 h. Identical banding pattern indicates the similarities in the protein composition within the cell across the selected mutants. M, Bio-Rad broad-range marker.

S2 Table. Protein homology of identified MH96 genes in selected gene clusters. Similarities of products of putative ORFs to translates amino acid sequence in the database detected using BLASTP.

Locus (Amino Acid Residues)	Closest BLASTp orthologues [Species]<br>**Length: Amino Acid (AA) Length of Orthologue**<br>%Identity/%AA Similarity (in Relation to) Target amino acid sequence	Accession Number
**YeRER cluster**		
PL78_17385 (138)	helix-turn-helix domain-containing protein [*Yersinia nurmii*]<br>**Length: 138**<br>96/98 (137) 1 -137	WP_049597149.1
	helix-turn-helix domain-containing protein [*Yersinia enterocolitica*]<br>**Length: 166**<br>35/64 (116) 45-162	WP_076706527.1
	Yen7 [*Yersinia entomophaga*]<br>**Length: 138**<br>59/77 (137) 1-137	ABG33871.1
	transcriptional regulatory protein phoP [*Yersinia enterocolitica* W22703]<br>**Length: 223**<br>26/59 (76) 147-219	CBX70398.1
PL78_17390 (104)	holin [*Yersinia nurmii*]<br>**Length: 104**<br>98/99 (104) 1-104	WP_049597150.1
	holin [*Rahnella* sp. AN3-3W3]<br>**Length: 102**<br>75/82 (102) 1-102	WP_112291644.1
	holin [*Yersinia enterocolitica* W22703]<br>**Length: 103**<br>58/72 (103) 1-103	CAI77377.1
PL78_17395 (129)	M15 family peptidase [*Yersinia nurmii*]<br>**Length: 129**<br>95/96 (129) 1-129	WP_049597151.1
	M15 family peptidase [*Erwinia amylovora*]<br>**Length: 129**<br>68/86 (129) 1-129	WP_004157153.1
	endolysin [*Yersinia enterocolitica* W22703]<br>**Length: 133**<br>69/79 (126) 8-133	CAI77378.1
PL78_17400 (133)	DUF2570 domain-containing protein [*Yersinia nurmii*]<br>**Length: 133**<br>93/94 (133) 1-133	WP_049597152.1
	hypothetical protein [*Chromobacterium* sp. ATCC 53434]<br>**Length: 121**<br>34/54 (124) 1-121	WP_101708791.1
	i-spanin [*Yersinia enterocolitica* W22703]<br>**Length: 119**<br>30/53 (93) 24-116	CAI77379.1
	o-spanin [*Yersinia enterocolitica* W22703]	SOH98281.1

Locus (Amino Acid Residues)	Closest BLASTp orthologues [Species] **Length: Amino Acid (AA) Length of Orthologue** %Identity/%AA Similarity (in Relation to) Target amino acid sequence	Accession Number
PL78_17400B (94)	**Length: 76**<br>43/52 (75) 2-75	
	unknown protein [*Yersinia enterocolitica* W22703]<br>**Length: 50**<br>46/54 (48) 2-49	CBX73607.1
**HNS cluster**		
PL78_04815 (214)	MULTISPECIES: YchE family NAAT transporter [*Yersinia*]<br>**Length: 214**<br>100/100 (214) 1 -214	WP_064513599.1
	YchE family NAAT transporter [*Yersinia nurmii*]<br>**Length: 214**<br>99/99 (214) 1-214	WP_049598201.1
PL78_04810 (890)	MULTISPECIES: bifunctional acetaldehyde-CoA/alcohol dehydrogenase [*Yersinia*]<br>**Length: 890**<br>100/100 (890) 1-890	WP_064513597.1
	bifunctional acetaldehyde-CoA/alcohol dehydrogenase [*Yersinia nurmii*]<br>**Length: 890**<br>99/99 (890) 1-890	WP_049598199.1
PL78_04805 (203)	MULTISPECIES: thymidine kinase [*Yersinia*]<br>**Length: 203**<br>100/100 (203) 1-203	WP_064513595.1
	thymidine kinase [*Yersinia nurmii*]<br>**Length: 203**<br>99/99 (203) 1-230	WP_049598197.1
PL78_04800 (135)	MULTISPECIES: DNA-binding transcriptional regulator H-NS [*Yersinia*]<br>**Length: 135**<br>100/100 (135) 1-135	WP_064513593.1
	MULTISPECIES: DNA-binding transcriptional regulator H-NS [*Dickeya*]<br>**Length: 135**<br>84/93 (135) 1-135	WP_013317859.1
PL78_04795 (449)	MULTISPECIES: UDP-glucose/GDP-mannose dehydrogenase family protein [*Yersinia*]<br>**Length: 449**<br>100/100 (449) 1-449	WP_064513591.1
	UDP-glucose/GDP-mannose dehydrogenase family protein [*Yersinia nurmii*]<br>**Length: 449**<br>99/99 (449) 1-449	WP_049598195.1
PL78_04790 (337)	two-component system response regulator RssB [*Yersinia entomophaga*]<br>**Length: 337**<br>100/100 (337) 1 -337	WP_064513589.1
	two-component system response regulator RssB [*Yersinia* sp. IP36721]<br>**Length: 337**<br>99/99 (337) 1-337	WP_120805445.1
PL78_04785	MULTISPECIES: patatin-like phospholipase RssA [*Yersinia*]	WP_064513588.1

Locus (Amino Acid Residues)	Closest BLASTp orthologues [Species]<br>**Length: Amino Acid (AA) Length of Orthologue**<br>%Identity/%AA Similarity (in Relation to) Target amino acid sequence	Accession Number
(309)	**Length: 309**<br>100/100 (309) 1 -309	
	patatin-like phospholipase RssA [*Yersinia nurmii*]<br>**Length: 309**<br>96/98 (309) 1-309	WP_049598191.1
**Yhf cluster**		
PL78_15425 (224)	MULTISPECIES: DUF1007 family protein [*Yersinia*]<br>**Length: 224**<br>100/100 (224) 1 -224	WP_064516843.1
	putative periplasmic or exported protein [*Yersinia nurmii*]<br>**Length: 224**<br>92/95 (224) 1-224	CNF17231.1
PL78_15420 (423)	MULTISPECIES: stationary phase inducible protein CsiE [*Yersinia*]<br>**Length: 423**<br>100/100 (423) 1-423	WP_064516841.1
	stationary phase inducible protein CsiE [*Yersinia nurmii*]<br>Length: 423<br>96/96 (423) 1-423	WP_049601759.1
PL78_15415 (385)	MULTISPECIES: 3-phenylpropionate MFS transporter [*Yersinia*]<br>**Length: 385**<br>100/100 (385) 1-385	WP_064516839.1
	3-phenylpropionate MFS transporter [*Yersinia nurmii*]<br>**Length: 385**<br>98/98 (385) 1-385	WP_049601760.1
PL78_15410 (417)	MULTISPECIES: serine hydroxymethyltransferase [*Yersinia*]<br>**Length: 417**<br>100/100 (417) 1-417	WP_064516837.1
	serine hydroxymethyltransferase [*Yersinia nurmii*]<br>**Length: 417**<br>99/100 (417) 1-417	WP_049601763.1
PL78_15405 (396)	MULTISPECIES: NO-inducible flavohemoprotein [*Yersinia*]<br>**Length: 396**<br>100/100 (396) 1-396	WP_064516835.1
	NO-inducible flavohemoprotein [*Yersinia nurmii*]<br>**Length: 396**<br>96/98 (396) 1-396	WP_049601766.1
PL78_15400 (299)	Multispecies: GntR family transcriptional regulator [*Yersinia*]<br>**Length: 299**<br>100/100 (299) 1 -299	WP_064516833.1
	GntR family transcriptional regulator [*Yersinia nurmii*]<br>**Length: 299**<br>99/100 (299) 1-299	WP_049601769.1
PL78_15395	MULTISPECIES: PRD domain-containing protein [*Yersinia*]	WP_064516831.1

Locus (Amino Acid Residues)	Closest BLASTp orthologues [Species] **Length: Amino Acid (AA) Length of Orthologue** %Identity/%AA Similarity (in Relation to) Target amino acid sequence	Accession Number
(122)	**Length: 122**<br>100/100 (122) 1-122	
	PRD domain-containing protein [*Yersinia nurmii*]<br>**Length: 122**<br>98/100 (122) 1-122	WP_049601772.1
PL78_15390 (384)	YhfX family PLP-dependent enzyme [*Yersinia* sp. IP36721]<br>**Length: 384**<br>99/100 (384) 1-384	WP_120806684.1
	YhfX family PLP-dependent enzyme [*Yersinia nurmii*]<br>**Length: 384**<br>95/98 (384) 1-384	WP_049601775.1
PL78_15385 (412)	MULTISPECIES: phosphopentomutase [*Yersinia*]<br>**Length: 412**<br>100/100 (412) 1-412	WP_064516827.1
	phosphopentomutase [*Yersinia nurmii*]<br>**Length: 412**<br>96/98 (412) 1-412	WP_049601777.1
PL78_15380 (295)	MULTISPECIES: phosphotriesterase-related protein [*Yersinia*]<br>**Length: 295**<br>100/100 (295) 1-295	WP_064516825.1
	phosphotriesterase-related protein [*Yersinia nurmii*]<br>**Length: 295**<br>98/99 (295) 1-295	WP_049601780.1
PL78_15375 (117)	MULTISPECIES: DUF2620 domain-containing protein [*Yersinia*]<br>**Length: 117**<br>100/100 (117) 1-117	WP_049601783.1
	DUF2620 domain-containing protein [*Yersinia frederiksenii*]<br>**Length: 117**<br>91/98 (117) 1-117	WP_1050108064.1
PL78_15370 (432)	MULTISPECIES: hypothetical protein [*Yersinia*]<br>**Length: 432**<br>100/100 (432) 1-432	WP_071925602.1
	membrane protein [*Yersinia nurmii*]<br>**Length: 432**<br>99/100 (432) 1-432	WP_049601784.1
PL78_15365 (112)	MULTISPECIES: nitrogen regulatory protein P-II [Yersiniaceae]<br>**Length: 112**<br>100/100 (112) 1-112	WP_004718065.1
	Nitrogen regulatory protein P-II [*Serratia marcescens*]<br>**Length: 112**<br>99/100 (112) 1-112	SAY44625.1
**RBS cluster**		

Locus (Amino Acid Residues)	Closest BLASTp orthologues [Species] **Length: Amino Acid (AA) Length of Orthologue** %Identity/%AA Similarity (in Relation to) Target amino acid sequence	Accession Number
PL78_12640 (502)	ATPase RavA [*Yersinia* sp. IP36721]<br>**Length: 502**<br>99/100 (502) 1-502	WP_120806543.1
	ATPase RavA [*Yersinia nurmii*]<br>**Length: 502**<br>97/99 (502) 1-502	WP_049597446.1
PL78_12645 (622)	MULTISPECIES: low affinity potassium transporter Kup [*Yersinia*]<br>**Length: 622**<br>100/100 (622) 1-622	WP_064516007.1
	low affinity potassium transporter Kup [*Yersinia nurmii*]<br>**Length: 622**<br>99/99 (622) 1-622	WP_049597447.1
PL78_12650 (139)	MULTISPECIES: D-ribose pyranase [*Yersinia*]<br>**Length: 139**<br>100/100 (139) 1-139	WP_049597449.1
	D-ribose pyranase [*Yersinia ruckeri*]<br>**Length: 139**<br>95/96 (139) 1-139	WP_038242232.1
PL78_12655 (510)	MULTISPECIES: ribose ABC transporter ATP-binding protein RbsA [*Yersinia*]<br>**Length: 510**<br>100/100 (510) 1-510	WP_064516010.1
	ribose ABC transporter ATP-binding protein RbsA [*Yersinia nurmii*]<br>**Length: 510**<br>99/99 (510) 1-510	WP_049597450.1
PL78_12660 (321)	MULTISPECIES: ribose ABC transporter permease [*Yersinia*]<br>**Length: 321**<br>100/100 (321) 1-321	WP_064516012.1
	ribose ABC transporter permease [*Yersinia nurmii*]<br>**Length: 321**<br>99/99 (321) 1-321	WP_049597451.1
PL78_12665 (296)	MULTISPECIES: ribose ABC transporter substrate-binding protein RbsB [*Yersinia*]<br>**Length: 296**<br>100/100 (296) 1-296	WP_064516014.1
	ribose ABC transporter substrate-binding protein RbsB [*Yersinia nurmii*]<br>**Length: 296**<br>99/100 (296) 1-296	WP_120805445
PL78_12670 (308)	ribokinase [*Yersinia* sp. IP36721]<br>**Length: 308**<br>99/100 (308) 1 -308	WP_120806544.1
	ribokinase [*Yersinia nurmii*]<br>**Length: 308**<br>97/99 (308) 1-308	WP_049597453.1

Locus (Amino Acid Residues)	Closest BLASTp orthologues [Species] **Length: Amino Acid (AA) Length of Orthologue** %Identity/%AA Similarity (in Relation to) Target amino acid sequence	Accession Number
PL78_12675 (333)	MULTISPECIES: ribose operon transcriptional repressor RbsR [*Yersinia*] **Length: 333** 100/100 (333) 1-333	WP_064516018.1
	ribose operon transcriptional repressor RbsR [*Yersinia ruckeri*] **Length: 337** 94/97 (333) 1-333	WP_038251067.1

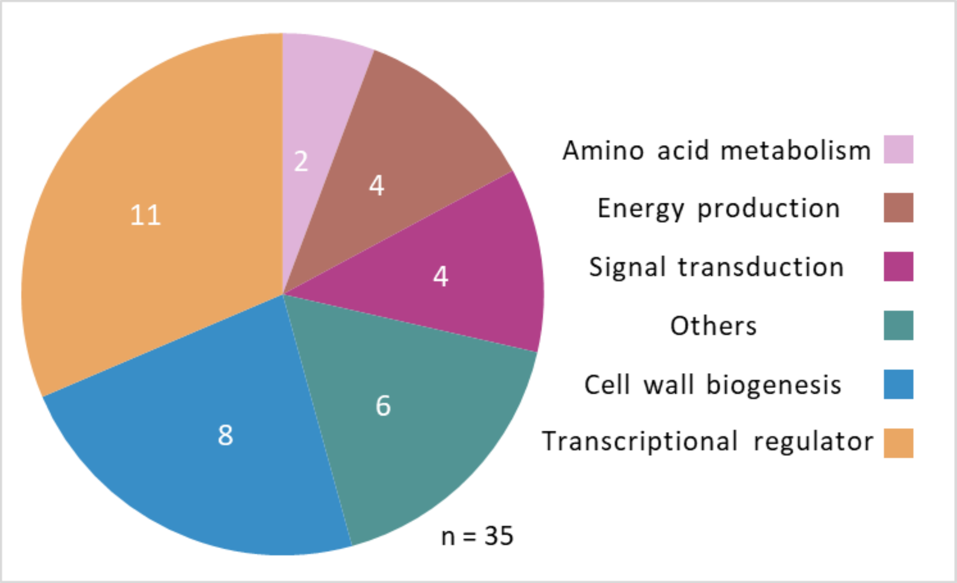

S2 Fig. Clusters of Orthologous Groups assigned to secretion deficient MH96 transposon mutants identified using high-throughput exoproteome screening assay. Transposon mutants and classification sourced from Table 1.

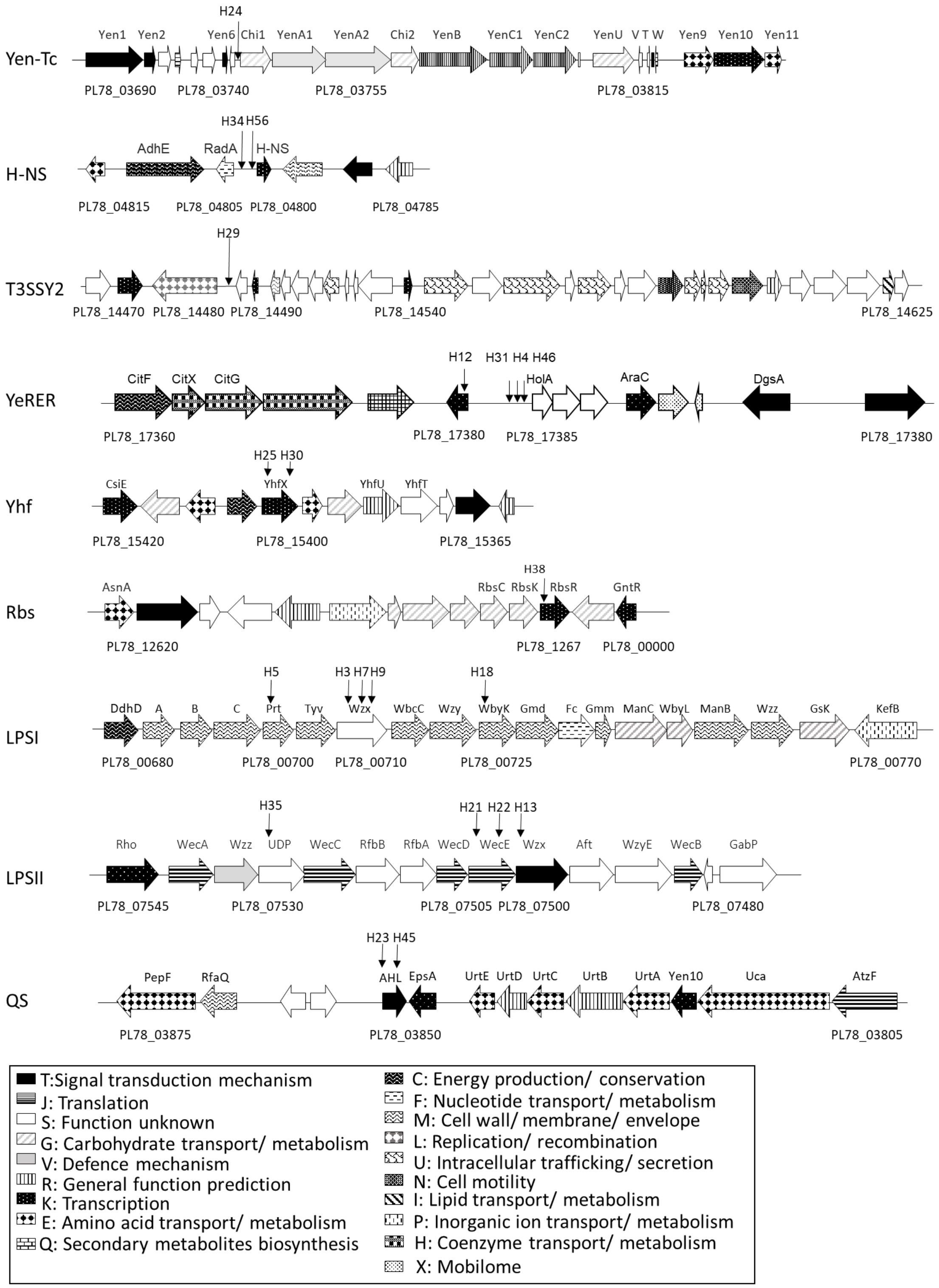

S3 Fig. Schematic depicting selected gene cluster affected by HESA MH96 transposon mutagenesis. Gene clusters with insertions within intergenic region, lipopolysaccharide clusters LPSI, LPSII and virulence associated gene clusters such as quorum sensing, Yen-Tc and T3SSY2. Vertical arrows denote MH96 transposon insertion points. Patterned horizontal arrows reflect COG classification as indicated in box.

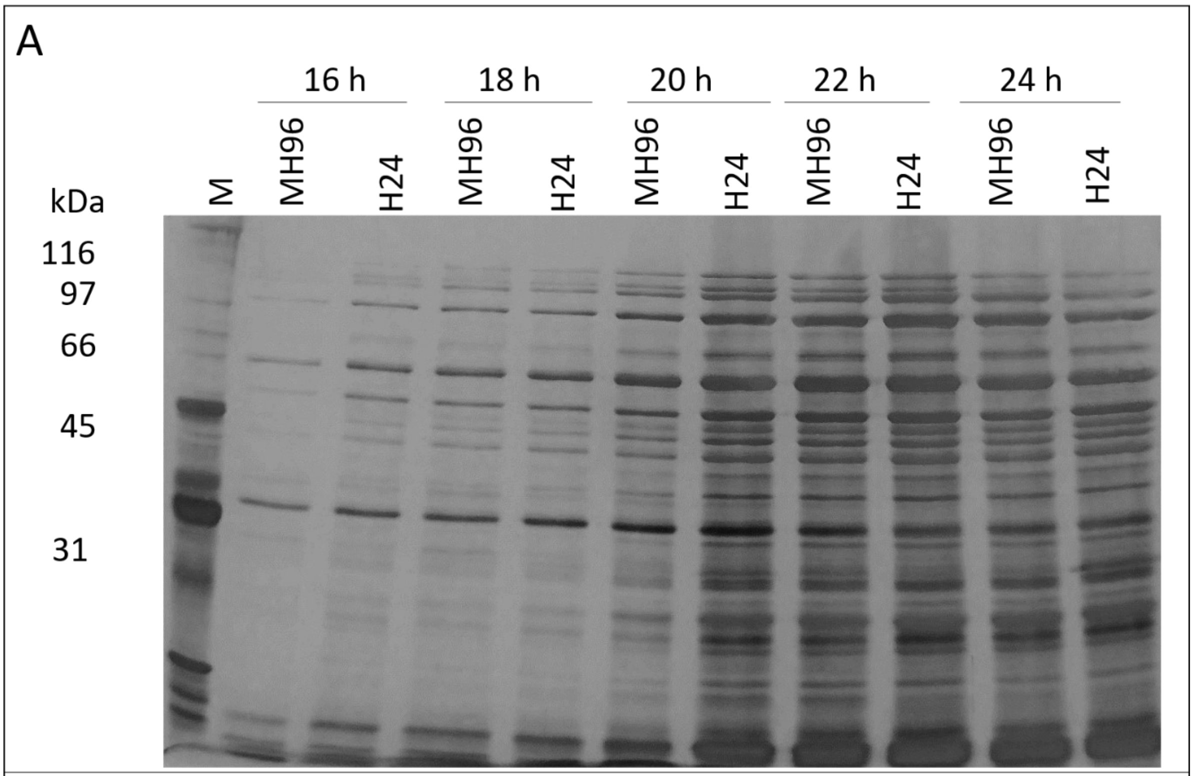

B

Time (h)	CFU (log10) MH96	CFU (log10) H24
16	3.60×10^9 (9.6)	4.35×10^9 (9.6)
18	3.35×10^9 (9.5)	3.00×10^9 (9.5)
20	3.45×10^9 (9.5)	2.95×10^9 (9.5)
22	4.90×10^9 (9.7)	4.60×10^9 (9.7)
24	3.50×10^9 (9.5)	2.85×10^9 (9.5)

S4 Fig. SDS-PAGE of exoprotein in the strains MH96 and H24 (*chi1-yen7* mutant) *of* 50-mL LB broth cultures. A) silver stained SDS-PAGE of MH96 and H24 of samples collected every 2 h from 16–24 hpi. Samples at each timepoint were taken from the same flask and assessed by SDS-PAGE. B) CFU log10 values of timepoints of MH96 and H24 cultures visualized in A, note the greater amount of exoprotein in H24 relative to MH96.
